# A Cross-Sectional Study on the Association Between Risk Factors of Toxoplasmosis and One Health Knowledge in Pakistan

**DOI:** 10.3389/fvets.2021.751130

**Published:** 2021-11-18

**Authors:** Tooba Maqsood, Khuram Shahzad, Shumaila Naz, Sami Simsek, Muhammad Sohail Afzal, Shahzad Ali, Haroon Ahmed, Jianping Cao

**Affiliations:** ^1^Department of Biosciences, COMSATS University Islamabad (CUI), Islamabad, Pakistan; ^2^Department of Biological Sciences, National University of Medical Sciences (NUMS), Rawalpindi, Pakistan; ^3^Department of Parasitology, Firat University, Elazig, Turkey; ^4^Department of Life Sciences, Faculty of Science, University of Management and Technology (UMT), Lahore, Pakistan; ^5^Department of Wildlife and Ecology, University of Veterinary and Animal Sciences, Lahore, Pakistan; ^6^National Institute of Parasitic Diseases, Chinese Center for Disease Control and Prevention (Chinese Center for Tropical Diseases Research), Shanghai, China; ^7^Key Laboratory of Parasite and Vector Biology, National Health Commission of People's Republic of China, Shanghai, China; ^8^World Health Organization (WHO) Collaborating Centre for Tropical Diseases, Shanghai, China; ^9^The School of Global Health, Chinese Center for Tropical Diseases Research, Shanghai Jiao Tong University School of Medicine, Shanghai, China

**Keywords:** knowledge, attitude, practices, risk factor, one health, toxoplasmosis, Pakistan

## Abstract

Toxoplasmosis is a zoonotic disease caused by *Toxoplasma gondii*, a protozoan that infects warm-blooded animals and humans. Approximately one third of the global population is infected by *T. gondii*. We conducted a cross-sectional study to assess the risk factors and One Health knowledge of toxoplasmosis in Rawalpindi and Islamabad, Pakistan. From July through December 2020, we collected data using questionnaires. The results showed that 60% of participants had heard or read about the disease, 23.3% of participants had no knowledge about the disease, and 16.8% participants were not sure about the disease. More than half of the participants (53.3%) reported that toxoplasmosis was caused by toxins, 5.3% reported that toxoplasmosis was an animal disease, 13.8% reported that toxoplasmosis was a human disease, 65.8% reported that it was both an animal and human disease, and 15.3% reported that it was neither an animal nor a human disease. Approximately 80.5% of participants reported that individuals acquired toxoplasmosis by changing cat litter. Our study findings revealed a low level of knowledge and awareness about toxoplasmosis among males. Therefore, there should be awareness programs to educate individuals about the risks of this deadly disease and to provide information on the major routes of transmission.

## Introduction

Toxoplasmosis is a zoonotic disease caused by the intracellular protozoan *Toxoplasma gondii* (1). *T. gondii* is an obligate intracellular parasite that naturally exists in one of three forms: (1) oocysts, which release sporozoites, are only produced in the small intestines of cats and are released into the environment through their feces; (2) tissue cysts, which release bradyzoites; and (3) tachyzoites, which are the proliferative form (2). Type I, II, and III strains of *T. gondii* have been identified in Europe, parts of Asia, and US where type II strain is mostly involved in human toxoplasmosis (3). Type I and type III strains are prevalent in Central and South America (4). Approximately 33% of the total human population has been affected by *T. gondii* (1). Countries in North America, Southeast Asia, Northern Europe, and Saharan African have low prevalence rates (10% to 30%), Central and Southern Europe have moderate prevalence rates (30% to 50%), and tropical African countries and Latin America have high prevalence rates of toxoplasmosis (5). The seroprevalence of toxoplasmosis was 29.45% from Southern Punjab, Pakistan (6). In Pakistan, Khyber Pakhtunkhwa has 40.6% of the seroprevalence of toxoplasmosis in women with poor obstetric history (7).

In humans, toxoplasmosis is transmitted by consuming raw or inadequately cooked meat (8), by inadvertently ingesting oocysts passed into feces by cats, either in a cat litter box or outdoors in the soil (9), and from mother to her unborn fetus (10). *T. gondii* infection, which is a life-threatening disease, results in retinal infection in both healthy and immunocompromised individuals (11). In immunocompromised individuals, toxoplasmosis is mostly asymptomatic (12); however, 10% of those infected may develop lymphadenitis, ocular toxoplasmosis (chorioretinitis), and mild flu-like and/or mononucleosis-like symptoms (13).

Due to their non-specificity, the clinical symptoms of toxoplasmosis are not reliable for diagnosis. While traditional diagnostic methods are based on serological tests and bioassays, a variety of molecular methods have been recently used for diagnosis of toxoplasmosis (14). Some of the diagnostic tests for toxoplasmosis include microscopy (15), bioassays (16), dye test (17), modified agglutination test (18), latex agglutination test (19), indirect hemagglutination test (20), indirect fluorescent antibody test (21), enzyme-linked immunosorbent assay (22), immunosorbent agglutination assay (23), immunochromatographic test (24), piezoelectric immunoagglutination assay (25), Western blot (26), and avidity test (27). Pharmaceutical interventions against toxoplasmosis include either a combination of pyrimethamine and sulfadiazine with folic acid or a combination of pyrimethamine and macrolide antibiotics or lincosamide. For congenital toxoplasmosis, pregnant women are treated with spiramycin (12).

Toxoplasmosis, which affects both animals and humans, causes major economic losses (28). In the livestock sector of Pakistan, different diseases cause annual economic loss of 79 billion Pakistani rupees (PKR) (29). Despite having such significant impact, very few studies have explored the prevalence of toxoplasmosis in Pakistan. Therefore, we conducted a study to determine the knowledge, attitudes, and practices of toxoplasmosis among university students of twin cities, Rawalpindi and Islamabad, Pakistan.

## Results

### Socio-Demographic Characteristics

Table 1 presents the sociodemographic characteristics of the participants (n = 400). Most of the participants (86%) were females. The majority of the participants (65.5%) were 18 to 25 years of age, 25.5% were 26 to 35 years of age, 5.5% were 36 to 45 years of age. Among the participants, 46.5% were from Punjabi, 15.3% were from Kashmiri, 7.8% were from Pathan, and 8% were from ethnicity. Approximately, 45% of the participants were in a Bachelor's program, 29.3% were in a master's program, 16.8% were in a PhD program.

**Table 1:** Socio-demographic characteristics of participants.

**Variable**	**Characteristics**	**Participants (*N*)**	**Frequency (%)**
Age (years)	18–25	262	65.5
	26–35	102	25.5
	36–45	22	5.5
	>45	14	3.5
Sex	Male	56	14.0
	Female	344	86.0
Ethnicity	Punjabi	186	46.5
	Sindhi	13	3.3
	Pathan	31	7.8
	Blochi	25	6.3
	Gilgiti	32	8.0
	Kashmiri	61	15.3
	Islamabad territory	35	8.8
	Other	17	4.3
Religion	Muslims	350	87.5
	Non-muslim	50	12.5
Marital status	Married	122	30.5
	Single	278	69.5
Education	Bachelors	180	45.0
	Master	117	29.3
	Ph.D.	67	16.8
	Post Doc	36	9.0
Occupation	Farmer (Household livestock)	68	17.0
	Worker at livestock facilities	40	10.0
	Other	292	73.0
Residence	Rural	142	35.5
	Urban	258	64.5
Income per month	<15,000 PKR (including pocket money)	151	37.7
	20,000–30,000 PKR	86	21.5
	>30,000 PKR	163	40.8
Number of family members	<5	79	19.8
	5–10	295	73.8
	11–15	26	6.5

### Knowledge on Toxoplasmosis

Among the participants, 60% had heard or read about the disease, 23.3% had no knowledge about the disease, and 16.8% were not sure about the disease. We performed Chi square test to assess the relationship among the categorical variables. Out of 400 participants, 53.3% reported that toxoplasmosis was caused by a toxin, 13.8% reported that toxoplasmosis was not caused by a toxin, and 33% had no knowledge on the cause of toxoplasmosis. Only a limited (19.3%) number of participants had been tested for toxoplasmosis, 67.5% of the participants were aware that toxoplasmosis was caused by an infection, and 26.3% reported that they had no knowledge on the causes of toxoplasmosis. The majority (69.8%) of the participants thought that a transmission source was cat feces, and 75.3% were aware that parasites were shed in the feces of infected cats. Approximately 58.5% of the participants reported that toxoplasmosis could be caused by touching raw meat and contaminated soil/sand and 26.8% of the participants had no knowledge about this. A significant number (68%) of participants reported that pregnant women could develop serious complications from toxoplasmosis, and 48.5% reported that the fetus and newborn could develop serious complications from toxoplasmosis. The majority (71.3%) of the participants reported that toxoplasmosis was transmitted from animals to humans, and 64% of the participants believed that toxoplasmosis is symptomatic. Approximately 47.5% of the participants reported that toxoplasmosis could cause miscarriages or stillbirth (Table 2).

**Table 2:** Knowledge of toxoplasmosis.

**Variable**	**Characteristics**	**Participants (*N*)**	**Frequency (%)**	**Statistical analysis (Chi Square)**
Have you heard or read about toxoplasmosis?	Yes	240	60.0	*X^2^*= 23.449 *df* = 2 *p* < 0.00001
	No	93	23.3	
	May be	67	16.8	
Can bacteria infect animals?	Yes	355	88.8	*X^2^* = 144.7348 *df* = 2 *p* < 0.00001
	No	14	3.5	
	Do not know	31	7.8	
Is toxoplasmosis caused by a toxin?	Yes	213	53.3	*X^2^* = 22.8448 *df* = 2 *p* = 0.000011
	No	55	13.8	
	Do not know	132	33.0	
Have you ever been tested for toxoplasmosis?	Yes	77	19.3	*X^2^* = 39.9216 *df* = 1 *p* < 0.00001
	No	323	80.8	
Is toxoplasmosis an infection?	Yes	270	67.5	*X^2^* = 65.4362 *df* = 2 *p* < 0.00001
	No	25	6.3	
	Do not know	105	26.3	
Is the parasite shed in the feces of infected cats?	Yes	301	75.3	*X^2^* = 99.0611 *df* = 2 *p* < 0.00001
	No	14	3.5	
	Do not know	85	21.3	
Is the parasite present in raw or undercooked meat?	Yes	311	77.8	*X^2^* = 103.7767 *df* = 2 *p* < 0.00001
	No	14	3.5	
	Do not know	75	18.8	
Is the parasite present in unpasteurized milk?	Yes	268	67.8	*X^2^* = 58.9505 *df* = 2 *p* < 0.00001
	No	29	7.3	
	Do not know	98	24.8	
Can individuals acquire toxoplasmosis by clean up the cat litter box?	Yes	279	69.8	*X^2^* = 65.1131 *df* = 2 *p* < 0.00001
	No	27	6.8	
	Do not know	94	23.5	
Can toxoplasmosis be caused by touching raw meat?	Yes	235	58.8	*X^2^* = 25.2688 *df* = 2 *p* < 0.00001
	No	58	14.5	
	Do not know	107	26.8	
Can individuals acquire toxoplasmosis by touching sand/soil in the garden or yard?	Yes	224	56.0	*X^2^* = 26.4293 *df* = 2 *p* < 0.00001
	No	52	13.0	
	Do not know	124	31.0	
Do pregnant women develop serious complications from toxoplasmosis?	Yes	272	68.0	*X^2^* = 87.0261 *df* = 2 *p* < 0.00001
	No	15	3.8	
	Do not know	113	28.2	
Do unborn and/or newborn children develop serious complications from toxoplasmosis?	Yes	251	62.7	*X^2^* = 57.6997 *df* = 2 *p* < 0.00001
	No	27	6.8	
	Do not know	122	30.5	
Does toxoplasmosis in a pregnant women cause fever and flu-like symptoms?	Yes	194	48.5	*X^2^* = 42.8536 *df* = 2 *p* < 0.00001
	No	33	8.3	
	Do not Know	173	43.3	
Does toxoplasmosis in pregnant women cause swollen glands?	Yes	160	40.0	*X^2^* = 46.3457 *df* = 2 *p* < 0.00001
	No	31	7.8	
	Do not know	209	52.3	
Can toxoplasmosis in pregnant women cause no symptoms?	Yes	153	38.3	*X^2^* = 11.4422 *df* = 2 *p* = 0.003276
	No	71	17.8	
	Do not know	176	44.0	
Can toxoplasmosis be transferred from a pregnant woman to her fetus if she became infected during her pregnancy?	Yes	186	46.5	*X^2^* = 17.4235 *df* = 2 *p* = 0.000165
	No	60	15.0	
	Do not know	154	38.5	
Can toxoplasmosis be transferred from a pregnant woman to her fetus if she became infected before her pregnancy?	Yes	164	41.0	*X^2^* = 10.0036 *df* = 2 *p* = 0.006726
	No	74	18.5	
	Do not know	162	40.5	
Can an infant with toxoplasmosis with no signs of illness at birth develop illness later in life?	Yes	218	54.5	*X^2^* = 40.3357 *df* = 2 *p* = 0.00001
	No	36	9.0	
	Do not know	146	36.5	
Can an infant with toxoplasmosis be treated with medicine?	Yes	209	52.3	*X^2^* = 49.4146 *df* = 2 *p* = 0.00001
	No	29	7.2	
	Do not know	162	40.5	
In which stage of gestation is toxoplasmosis highly severe?	First	31	7.8	*X^2^* = 38.1639 *df* = 3 *p* = 0.00001
	Second	56	14.0	
	Third	117	29.3	
	Do not know	196	49.0	
Are you aware that pregnant women should not smoke?	Yes	317	79.3	*X^2^* = 96.641 *df* = 2 *p* < 0.00001
	No	19	4.8	
	Do not know	64	16.0	
Can women who have toxoplasmosis before they get pregnant transmit it to the baby?	Yes	195	48.8	*X^2^* = 15.8341 *df* = 2 *p* = 0.000364
	No	64	16.0	
	Do not know	141	35.3	
Can toxoplasmosis be treated in pregnant women?	Yes	248	62.0	*X^2^* = 60.5088 *df* = 2 *p* < 0.00001
	No	25	6.3	
	Do not know	127	31.8	
Do infants with toxoplasmosis develop vision problems?	Yes	238	59.5	*X^2^* = 48.0302 *df* = 2 *p* < 0.00001
	No	32	8.0	
	Do not know	130	32.5	
Should cat litter be replaced daily?	Yes	283	70.8	*X^2^* = 59.0899 *df* = 2 *p* < 0.00001
	No	33	8.3	
	Do not know	84	21.0	
Can pregnant women avoid toxoplasmosis by consuming thoroughly cooked meat?	Yes	263	65.8	*X^2^* = 57.3761 *df* = 2 *p* < 0.00001
	No	29	7.2	
	Do not know	108	27.0	
Can individuals avoid toxoplasmosis by washing and peeling all fruits and vegetables before consumption?	Yes	270	67.5	*X^2^* = 59.3376 *df* = 2 *p* < 0.00001
	No	29	7.2	
	Do not know	101	25.3	
Which is the diagnostic method of toxoplasmosis in the fetus?	Ultrasound	244	61.0	*X^2^* = 23.6343 *df* = 2 *p* < 0.00001
	CT Scan	77	19.3	
	Do not know	79	19.8	
Toxoplasma gondii is a	Bacterium	37	9.3	*X^2^* = 125.594 *df* = 5 *p* = 5
	Virus	46	11.5	
	Parasite	221	55.3	
	Fungi	7	1.8	
	Insect	1	0.3	
	I am not sure	88	22.0	
Can toxoplasmosis be transmitted from animals to humans?	Yes	285	71.3	*X^2^* = 88.1145 *df* = 2 *p* < 0.00001
	No	16	4.0	
	Do not know	99	24.8	
Is toxoplasmosis associated with symptoms?	Yes	256	64.0	*X^2^* = 37.3477 *df* = 2 *p* < 0.00001
	No	47	11.8	
	Do not know	97	24.3	
Does toxoplasmosis affect only pregnant women?	Yes	154	38.5	*X^2^* = 13.9087 *df* = 2 *p* = 0.000954
	No	180	45.0	
	Do not know	66	16.5	
Does toxoplasmosis cause miscarriage or stillbirth?	Yes	190	47.5	*X^2^* = 10.4531 *df* = 2 *p* = 0.005372
	No	76	19.0	
	Do not know	134	33.5	

### Attitudes Toward Toxoplasmosis

Among the participants, 5.3% believed that toxoplasmosis was an animal disease, 13.8% thought that toxoplasmosis was a human disease, 65.8% thought it was both an animal and human disease, and 15.3% thought it was not either of them. Most of the participants (87.3%) routinely washed their hands after gardening. Approximately, 85.8% of the participants washed their hands after changing the cat litter and after handling raw meat. Most of the participants (89%) cooked meat wel-prior to consumption, and 86.3% avoided raw milk. A significant number of participants (86.5%) reported consuming untreated water, and the majority (80%) of the participants considered toxoplasmosis to be a dangerous disease. A small number (33.5%) of participants had consumed undercooked meat, and 64.5% had direct contact with cats. Approximately, 56.7% of participants had attended training related to livestock. A significant number (83%) of participants supported initiatives for the control of toxoplasmosis. There were no significant differences in the results when asked how health should be ensured when buying or receiving new livestock. Approximately 55% of the participants thought that toxoplasmosis-suspected cases should seek the advice of healthcare providers (Table 3).

**Table 3:** Attitudes toward toxoplasmosis.

**Variable**	**Characteristics**	**Participants (*N*)**	**Frequency (%)**	**Statistical analysis (Chi Square)**
What is your perception about toxoplasmosis?	Serious animal disease	21	5.3	*X^2^* = 67.7315 *df* = 3 *p* < 0.00001
	Serious human disease	55	13.8	
	Both	263	65.8	
	None	61	15.3	
Do you routinely wash your hands after gardening?	Yes	349	87.3	*X^2^* = 68.8634 *df* = 1 *p* < 0.00001
	No	51	12.7	
Do you routinely wash your hands after changing the cat litter box?	Yes	343	85.8	*X^2^* = 60.7863 *df* = 1 *p* < 0.00001
	No	57	14.2	
Do you routinely wash your hands after handling raw meat?	Yes	343	85.8	*X^2^* = 60.7863 *df* = 1 *p* < 0.00001
	No	57	14.2	
Do you thoroughly cook meat before consumption?	Yes	356	89.0	*X^2^* = 79.7086 *df* = 1 *p* < 0.00001
	No	44	11.0	
Do you avoid consuming raw milk?	Yes	345	86.3	*X^2^* = 63.3665 *df* = 1 *p* < 0.00001
	No	55	13.7	
Do you avoid consuming untreated water?	Yes	346	86.5	*X^2^* = 64.6975 *df* = 1 *p* < 0.00001
	No	54	13.5	
Is toxoplasmosis dangerous?	Yes	320	80.0	*X^2^* = 89.0025 *df* = 2 *p* < 0.00001
	No	26	6.5	
	Do not know	54	13.5	
Can toxoplasmosis be transmitted by consuming inadequately washed vegetables and undercooked meat?	Yes	308	77.0	*X^2^* = 71.8426 *df* = 2 *p* < 0.00001
	No	35	8.8	
	Do not know	57	14.2	
Do you consume undercooked meat?	Yes	134	33.5	*X^2^* = 9.3647 *df* = 1 *p* = 0.002212
	No	266	66.5	
Do you have direct contact with a cat?	Yes	258	64.5	*X^2^* = 7.1107 *df* = 1 *p* = 0.007662
	No	142	35.5	
Are fruits and vegetables in contact with cat feces?	Yes	200	50.0	*X^2^* = 8.8076 *df* = 2 *p* = 0.012231
	No	110	27.5	
	Do not know	90	22.5	
Do you wash kitchen utensils after contact with raw meat and unwashed fruits and vegetables?	Yes	327	81.8	*X^2^* = 43.4781 *df* = 1 *p* < 0.00001
	No	73	18.2	
Do you wear personal protective equipment while handling your cat?	Yes	258	64.5	*X^2^* = 7.1107 *df* = 1 *p* = 0.007662
	No	142	35.5	
Have you attended any training, awareness session or workshop related to livestock?	Yes	173	43.3	*X^2^* = 1.4752 *df* = 1 *p* = 0.224526 not significant
	No	227	56.7	
Will you support any initiative to control toxoplasmosis?	Yes	332	83.0	*X^2^* = 48.3184 *df* = 1 *p* < 0.00001
	No	68	17.0	
Does toxoplasmosis affect the production of livestock?	Yes	281	70.3	*X^2^* = 55.2524 *df* = 2 *p* < 0.00001
	No	36	9.0	
	Do not know	83	20.7	
How can health be ensured when buying or receiving new livestock?	Seek veterinary advice	139	34.8	*X^2^* = 6.0507 *df* = 3 *p* = 0.109168 not significant
	Rely on own experience	68	17.0	
	Acquire from known and/or trusted people	111	27.7	
	None	82	20.5	
What should an individual with suspected toxoplasmosis do?	Pray	34	8.5	*X^2^* = 37.2797 *df* = 3 *p* < 0.00001
	Visit health facility	220	55.0	
	Consuming herbal products	66	16.5	
	Visit local chemist and acquire medicine	80	20.0	

### Practices Toward Toxoplasmosis

Most of the participants (71%) fed their cats dry or commercial cat food and did not let their cats kill and eat rodents. Approximately, 61.8% of the participants reported that they avoided stray cats, and 70% of the participants did not allow someone else change the cat litter box. A significant number (83.3%) of participants boiled milk before consumption, and 87.8% ensured their houses were free of waste. Most participants (86.5%) kept foods covered in containers, and 78.3% separated sick animals from healthy ones. A significant number (72.8%) of participants used protective clothes while handling livestock (Table 4).

**Table 4:** Practices toward toxoplasmosis.

**Variable**	**Characteristics**	**Participants (*N*)**	**Frequency (%)**	**Statistical analysis (Chi Square)**
Do you feed your cat dry or commercial food and not let it kill and eat rodents?	Yes	284	71.0	*X^2^* = 15.9079 *df* = 1 *p* = 0.000066
	No	116	29.0	
Do you avoid stray cats?	Yes	247	61.8	*X^2^* = 4.5799 *df* = 1 *p* = 0.03235
	No	153	38.2	
Do you let someone else change the cat litter box?	Yes	280	70.0	*X^2^* = 14.2602 *df* = 1 *p* = 0.000159
	No	120	30.0	
Do you change the cat litter box daily?	Yes	323	80.8	*X^2^* = 39.9216 *df* = 1 *p* < 0.00001
	No	77	19.2	
Do you have a vegetable garden at home?	Yes	275	68.8	*X^2^* = 12.3626 *df* = 1 *p* = 0.000438
	No	125	31.2	
Do you boil milk before consumption?	Yes	333	83.3	*X^2^* = 49.3434 *df* = 1 *p* < 0.00001
	No	67	16.7	
Do you ensure that your house is free of waste?	Yes	351	87.8	*X^2^* = 71.7934 *df* = 1 *p* < 0.00001
	No	49	12.2	
Do you store food in covered containers?	Yes	346	86.5	*X^2^* = 64.6975 *df* = 1 *p* < 0.00001
	No	54	13.5	
Do you keep newly purchased animals in quarantine for some time?	Yes	236	59.0	*X^2^* = 2.6469 *df* = 1 *p* = 0.103753 not significant
	No	164	41.0	
Do you separate sick animals from healthy animals?	Yes	313	78.3	*X^2^* = 32.0952 *df* = 1 *p* < 0.00001
	No	87	21.7	
Do you use any kind of protective clothing while handling livestock?	Yes	291	72.8	*X^2^* = 19.0916 *df* = 1 *p* = 0.000012
	No	109	27.2	

### Risk Factors Associated With Toxoplasmosis

A significant number (80.5%) of participants thought that individuals could acquire toxoplasmosis by changing the cat litter, and 76.8% of participants responded that individuals could acquire toxoplasmosis by consuming raw/undercooked meat. Most participants (72.5%) believed that individuals could get toxoplasmosis by consuming raw milk, while 14.2% were not aware of this. Among the participants, 69.5% considered blood transfusion to be cause of toxoplasmosis, 13.7% considered that blood transfusion was not a cause of toxoplasmosis, and 16.8% had no knowledge about this. A significant number (68.3%) of participants thought toxoplasmosis could be transmitted by gardening without gloves. Among the participants, 47% believed that immunocompromised, pregnant women were at high risk of toxoplasmosis, 20.3% believed that pregnant women had a moderate risk of toxoplasmosis, 6.5% people reported that pregnant women had a low risk of toxoplasmosis, and 26.2% had no knowledge (Table 5).

**Table 5:** Risk factors of toxoplasmosis.

**Variable**	**Characteristics**	**Participants (*N*)**	**Frequency (%)**	**Statistical analysis (Chi Square)**
Can individuals acquire toxoplasmosis by changing cat litter?	Yes	322	80.5	*X^2^* = 39.073 *df* = 1 *p* < 0.00001
	No	78	19.5	
Can individuals acquire toxoplasmosis by consuming raw/undercooked meat?	Yes	307	76.8	*X^2^* = 67.7466 *df* = 2 *p* < 0.00001
	No	43	10.7	
	Do not know	50	12.5	
Can individuals acquire toxoplasmosis by consuming raw milk?	Yes	290	72.5	*X^2^* = 52.0668 *df* = 2 *p* < 0.00001
	No	53	13.3	
	Do not know	57	14.2	
Can individuals acquire toxoplasmosis by consuming raw vegetables?	Yes	276	69.0	*X^2^* = 41.6118 *df* = 2 p < 0.00001
	No	63	15.8	
	Do not know	61	15.2	
Can individuals acquire toxoplasmosis through blood transfusions?	Yes	278	69.5	*X^2^* = 43.5633 *df* = 2 *p* < 0.00001
	No	55	13.7	
	Do not know	67	16.8	
Can individuals acquire toxoplasmosis by consuming untreated water?	Yes	286	71.5	*X^2^* = 48.9029 *df* = 2 *p* < 0.00001
	No	55	13.7	
	Do not know	59	14.8	
Can individuals acquire toxoplasmosis by gardening without gloves?	Yes	273	68.3	*X^2^* = 40.4536 *df* = 2 *p* < 0.00001
	No	56	14.0	
	Do not know	71	17.7	
What is the risk level of toxoplasmosis among immunocompromised, pregnant women?	High	188	47.0	*X^2^* = 36.8318 *df* = 3 *p* < 0.00001
	Medium	81	20.3	
	Low	26	6.5	
	Do not know	105	26.2	

### One Health Knowledge of Toxoplasmosis

The majority (61.5%) of the participants knew about One Health, and 14% had no knowledge about One Health. Approximately 60.3% of participants knew about zoonosis, 26.5% were not aware of zoonosis, and 13.2% of participants were not sure about the concept of zoonosis. Out of 400 participants, 232 (58%) knew that toxoplasmosis is a zoonotic infection, and 18 participants (4.5%) had no knowledge on this. Only 8.8% of participants reported that toxoplasmosis was present in humans, 11% people reported that toxoplasmosis was present in livestock, 62.7% reported that toxoplasmosis was present in both humans and livestock, while 17.5% were not sure about this. Among the participants, 37.3% thought that toxoplasmosis causes blindness, 23.2% thought that toxoplasmosis did not cause blindness, and 39.5% were not sure about this (Table 6).

**Table 6:** One health knowledge about toxoplasmosis among participants.

**Variable**	**Characteristics**	**Participants (*N*)**	**Frequency (%)**	**Statistical analysis (Chi Square)**
Do you know about one health?	Yes	246	61.5	*X^2^* = 29.2344 *df* = 2 *p* < 0.00001
	No	56	14.0	
	Not sure	98	24.5	
Do you have knowledge on zoonosis?	Yes	241	60.3	*X^2^* = 29.381 *df* = 2 *p* < 0.00001
	No	106	26.5	
	Not sure	53	13.2	
Is toxoplasmosis a zoonotic disease?	Yes	232	58.0	*X^2^* = 72.8102 *df* = 2 *p* < 0.00001
	No	18	4.5	
	Not sure	150	37.5	
How is toxoplasmosis transmitted?	Soil	10	2.5	*X^2^* = 93.52 *df* = 5 *p* = 0
	Water	12	3.0	
	Livestock	56	14.0	
	a and b	21	5.3	
	a and c	224	56	
	Not sure	77	19.2	
Which organisms does toxoplasmosis affect?	Human	35	8.8	*X^2^* = 51.988 *df* = 3 *p* < 0.00001
	Livestock	44	11.0	
	Both human and livestock	251	62.7	
	Not sure	70	17.5	
Does toxoplasmosis go away?	Yes	211	52.8	*X^2^* = 54.5757 *df* = 2 *p* < 0.00001
	No	26	6.5	
	Not sure	163	40.7	
Can toxoplasmosis-infected individuals go blind?	Yes	149	37.3	*X^2^* = 4.1335 *df* = 2 *p* = 0.126594 not significant
	No	93	23.2	
	Not sure	158	39.5	

### Association Among Different Variables Based on ANOVA

We used one-way ANOVA to determine whether there were any statistically significant differences among the means of three or more independent groups. We used six specific independent variables, i.e., age, sex, ethnicity, education, religion, and marital status, and five dependent variables, i.e., knowledge, attitudes, practices, risk factors, and One Health. Our ANOVA results revealed that age was associated (*p* < 0.05) with attitudes and One Health; however, there were no significant associations with sex. Ethnicity was associated (*p* < 0.05) with knowledge and One Health; religion was associated (*p* < 0.05) with One health; and marital status was associated (*p* < 0.05) with knowledge, attitudes, risk factors, and One health. Likewise, the education of the participants was associated (*p* < 0.05) with knowledge, risk factors, and One Health (Table 7).

**Table 7:** Association between demographic characteristics and knowledge, attitude, practices, one health, and risk factors (ANOVA).

**Variable**	**Knowledge**				**Attitude**				**Practices**				**Risk factors**				**One health**			
	**M**	**SD**	**SE**	***P*-value**	**M**	**SD**	**SE**	***P*-value**	**M**	**SD**	**SE**	***P*-value**	**M**	**SD**	**SE**	***P*-value**	**M**	**SD**	**SE**	***P*-value**
**Age (in years)**																				
18–25	18.48	9.00	0.556	0.097	13.26	3.44	0.212	0.009	8.33	2.60	0.161	0.090	5.46	2.70	0.167	0.472	3.59	2.42	0.150	0.006
26–35	21.04	9.38	0.929		13.24	3.90	0.386		8.25	2.73	0.270		5.87	2.78	0.275		4.37	2.57	0.255	
36–45	20.36	10.97	2.339		11.27	4.76	1.015		7.09	3.94	0.840		5.59	3.26	0.695		4.18	2.84	0.605	
above 45	21.00	11.57	3.092		10.71	5.06	1.352		7.07	3.54	0.946		4.86	3.51	0.937		5.36	2.92	0.782	
**Gender**																				
Female	19.38	9.37	0.505	0.769	13.15	3.68	0.198	0.219	8.30	2.69	0.145	0.060	5.60	2.73	0.147	0.414	3.92	2.50	0.135	0.441
Male	18.98	9.27	1.239		12.48	4.09	0.547		7.55	3.17	0.424		5.27	3.11	0.416		3.64	2.76	0.369	
**Ethnicity**																				
Islamabad territory	22.14	7.87	1.330	0.009	13.66	3.23	0.545	0.313	8.66	2.62	0.443	0.664	6.40	2.45	0.414	0.220	4.74	2.33	0.394	<0.001
Kashmiri	16.62	7.75	0.993		12.82	4.08	0.522		8.11	2.07	0.266		4.87	2.60	0.333		2.82	1.94	0.248	
Punjabi	19.13	9.40	0.689		13.44	3.31	0.243		8.25	2.56	0.188		5.77	2.67	0.196		3.93	2.61	0.192	
Blochi	20.12	11.81	2.362		11.60	5.16	1.031		7.64	3.85	0.770		5.48	3.51	0.703		4.80	2.69	0.539	
Gilgiti	23.47	9.48	1.676		12.59	4.25	0.751		7.81	3.59	0.634		5.25	3.03	0.535		5.22	2.38	0.421	
Other	15.35	7.87	1.908		12.53	2.60	0.631		8.29	1.53	0.371		5.29	2.20	0.534		2.35	1.62	0.392	
Pathan	19.03	10.64	1.911		12.68	4.63	0.831		7.87	4.04	0.725		5.10	3.40	0.611		3.32	2.68	0.481	
Sindhi	21.31	7.28	2.020		12.54	3.45	0.958		9.23	1.48	0.411		5.54	2.63	0.730		4.23	2.20	0.611	
**Religion**																				
Muslim	18.93	9.40	0.503	0.024	13.02	3.72	0.199	0.646	8.09	2.77	0.148	0.048	5.55	2.77	0.148	0.935	3.77	2.52	0.135	0.013
Non-muslim	22.10	8.49	1.201		13.28	3.96	0.560		8.92	2.65	0.375		5.52	2.93	0.414		4.72	2.47	0.349	
**Marital status**																				
Married	21.54	9.85	0.891	0.002	12.43	4.49	0.406	0.026	8.20	2.99	0.271	0.997	6.00	2.75	0.249	0.032	4.69	2.53	0.229	<0.001
Single	18.35	8.96	0.537		13.33	3.34	0.200		8.20	2.67	0.160		5.35	2.78	0.167		3.53	2.46	0.147	
**Qualification**																				
Bachelors	16.33	8.43	0.629	<0.001	12.89	3.83	0.285	0.565	8.31	2.57	0.192	0.870	5.02	2.72	0.203	0.005	3.07	2.22	0.166	<0.001
Masters	20.21	9.70	0.896		12.91	3.82	0.353		8.04	2.80	0.259		5.84	2.72	0.252		3.98	2.65	0.245	
Ph.D	22.85	8.79	1.074		13.58	3.54	0.433		8.13	3.08	0.376		6.15	2.84	0.346		4.99	2.42	0.296	
Post doc	24.83	8.42	1.404		13.33	3.46	0.576		8.25	3.06	0.511		6.17	2.79	0.465		5.58	2.22	0.370	

### Statistical Analysis Using Log-Linear Regression

Log-linear regression analysis involves using a dependent variable measured by frequency counts with categorical or continuous independent predictor variable. Log-linear analysis is a technique used in statistics to examine the relationship between more than two categorical variables. The technique is used for both hypothesis testing and model building. In this study, we used the independent variables age, gender, ethnicity, education, religion, and marital status and the dependent variables knowledge, attitudes, practices, risk factors, and One Health. We applied log-linear regression on age and the dependent variables and obtained different *p*-values, rate ratios, and *R*^*2*^_McF_. Mostly high *R*^*2*^_McF_ values represent goodness of fit. With the dependent variable knowledge, we obtained *p* < 0.001, a rate ratio of 18.48, and an *R*^*2*^_McF_ value of 0.0124. With attitude as the dependent variable, we obtained *p* < 0.001, a rate ratio of 13.256, and an *R*^*2*^_McF_ value of 0.0241. With the dependent variable practices, we obtained *p* < 0.001, a rate ratio of 8.332, and an *R*^*2*^_McF_ value 0.0119. With risk factors, we obtained *p* < 0.001, a rate ratio of 5.458, and an *R*^*2*^_McF_ value of 0.00430. Finally, with One Health, we obtained *p* < 0.001, a rate ratio of 3.59, and an *R*^*2*^_McF_ value of 0.0220. The highest and lowest *R*^*2*^_McF_ values were obtained for attitudes and risk factors, respectively.

We applied a log-linear regression on the independent variable gender and the dependent variables. With knowledge, we obtained *p* < 0.001, a rate ratio of 19.378, and an *R*^*2*^_McF_ value of 1.73e-4. With attitudes, we obtained *p* < 0.001, a rate ratio of 13.145, and an *R*^*2*^_McF_ value of 0.00307. With practices, we obtained *p* < 0.001, a rate ratio of 8.302, and an *R*^*2*^_McF_ value of 0.00633. With risk factors, we obtained *p* < 0.001, a rate ratio of 5.596, and an *R*^*2*^_McF_ value of 0.00115. With One Health, we obtained *p* < 0.001, a rate ratio of 3.924, and an *R*^*2*^_McF_ value of 0.00113. The highest and lowest *R*^*2*^_McF_ values were obtained for knowledge and One Health, respectively.

We applied log-linear regression on the independent variable ethnicity and the dependent variables. With knowledge, we obtained *p* < 0.001, a rate ratio of 22.143, and an *R*^*2*^_McF_ value of 0.0367. With attitudes, we obtained *p* < 0.001, a rate ratio of 13.657, and an *R*^*2*^_McF_ value of 0.0168. With practices, we obtained *p* < 0.001, a rate ratio of 8.657, and an *R*^*2*^_McF_ value of 0.00868. With risk factors, we obtained *p* < 0.001, a rate ratio of 6.400, and an *R*^*2*^_McF_ value of 0.0161. With One Health, we obtained *p* < 0.001, a rate ratio of 4.743, and an *R*^*2*^_McF_ value of 0.0672. The highest and lowest *R*^*2*^_McF_ values were for One Health and practices, respectively (Table 8).

**Table 8:** Association between demographic characteristics and knowledge, attitudes, practices, one health, and risk factors (log-linear regression).

	**Knowledge**				**Attitude**				**Practices**				**Risk factor**				**One health**			
**Predictor**	**95% CI (Lower–upper)**	**Rate ratio**	***P***	***R* ^**2**^_**McF**_**	**95% CI (Lower–upper)**	**Rate ratio**	***P***	***R* ^**2**^_**McF**_**	**95% CI (Lower–upper)**	**Rate ratio**	***P***	***R* ^**2**^_**McF**_**	**95% CI (Lower–upper)**	**Rate ratio**	***P***	***R* ^**2**^_**McF**_**	**95% CI (Lower–upper)**	**Rate ratio**	***P***	***R* ^**2**^_**McF**_**
Intercept	Lower 2.8884 Upper 2.945	18.48	<0.001		Lower 2.5512 Upper 2.6177	13.256	<0.001		Lower 2.0782 Upper 2.16206	8.332	<0.001		Lower 1.6453 Upper 1.749	5.458	<0.001		Lower 1.2147 Upper 1.342	3.59	<0.001	
**Age (in years)**																				
26–35–18–25	Lower 0.0790 Upper 0.181	1.14	<0.001	0.0124	Lower −0.0644 Upper −0.0613	0.998	0.962	0.0241	Lower −0.0900 Upper 0.06905	0.990	0.796	0.0119	Lower −0.0222 Upper 0.169	1.076	0.133	0.00430	Lower 0.0841 Upper 0.309	1.22	<0.001	0.0220
36–45–18–25	Lower 4.33e-4 Upper 0.194	1.10	0.049		Lower −0.2909 Upper −0.0332	0.850	0.014		Lower −0.3237 Upper 0.00114	0.851	0.052		Lower −0.1601 Upper 0.208	1.024	0.798		Lower −0.0619 Upper 0.366	1.16	0.164	
Above 45–18–25	Lower 0.0103 Upper 0.246	1.14	0.033		Lower −0.3763 Upper −0.0494	0.808	0.011		Lower −0.3654 Upper 0.03735	0.849	0.110		Lower −0.3599 Upper 0.127	0.890	0.347		Lower 0.1647 Upper 0.635	1.49	<0.001	
Intercept	Lower 2.9401 Upper 2.9881	19.378	<0.001		Lower 2.547 Upper 2.6052	13.145	<0.001		Lower 2.080 Upper 2.15321	8.302	<0.001		Lower 1.677 Upper 1.7667	5.596	<0.001		Lower 1.314 Upper 1.4206	3.924	<0.001	
**Gender**																				
Male–female	Lower −0.0854 Upper 0.0441	0.980	0.532	1.73e-4	Lower −0.131 Upper 0.0279	0.950	0.203	0.00307	Lower −0.197 Upper 0.00759	0.910	0.070	0.00633	Lower −0.183 Upper 0.0621	0.941	0.334	0.00115	Lower −0.222 Upper 0.0728	0.928	0.322	0.00113
Intercept	Lower 3.0271 Upper 3.1679	22.143	<0.001		Lower 2.525 Upper 2.7039	13.657	<0.001		Lower 2.046 Upper 2.2710	8.657	<0.001		Lower 1.725 Upper 1.98725	6.400	<0.001		Lower 1.405 Upper 1.7088	4.743	<0.001	
**Ethnicity**																				
Kashmiri–Islamabad territory	Lower −0.3802 Upper −0.1932	0.751	<0.001	0.0367	Lower −0.177 Upper 0.0505	0.939	0.276	0.0168	Lower −0.208 Upper 0.0783	0.937	0.375	0.00868	Lower −0.447 Upper −0.09999	0.761	0.002	0.0161	Lower −0.733 Upper −0.3068	0.595	<0.001	0.0672
Punjabi–Islamabad territory	Lower −0.2240 Upper −0.0686	0.864	<0.001		Lower −0.114 Upper 0.0815	0.984	0.743		Lower −0.171 Upper 0.0754	0.953	0.447		Lower −0.247 Upper 0.04107	0.902	0.161		Lower −0.356 Upper −0.0195	0.829	0.029	
Blochi–Islamabad territory	Lower −0.2080 Upper 0.0164	0.909	0.094		Lower −0.309 Upper −0.0174	0.849	0.028		Lower −0.306 Upper 0.0561	0.883	0.176		Lower −0.368 Upper 0.05738	0.856	0.152		Lower −0.223 Upper 0.2468	1.012	0.920	
Gilgiti–Islamabad territory	Lower −0.0422 Upper 0.1585	1.060	0.256		Lower −0.214 Upper 0.0515	0.922	0.231		Lower −0.270 Upper 0.0648	0.902	0.230		Lower −0.398 Upper 0.00197	0.820	0.052		Lower −0.119 Upper 0.3104	1.100	0.383	
0ther–Islamabad territory	Lower −0.5065 Upper −0.2259	0.693	<0.001		Lower −0.248 Upper 0.0753	0.917	0.295		Lower −0.243 Upper 0.1570	0.958	0.674		Lower −0.434 Upper 0.05490	0.827	0.129		Lower −1.046 Upper −0.3558	0.496	<0.001	
Pathan–Islamabad territory	Lower −0.2585 Upper −0.0443	0.860	0.006		Lower −0.208 Upper 0.0590	0.928	0.274		Lower −0.264 Upper 0.0734	0.909	0.268		Lower −0.431 Upper −0.02407	0.796	0.028		Lower −0.602 Upper −0.1101	0.701	0.005	
Sindhi–Islamabad territory	Lower −0.1757 Upper 0.0988	0.962	0.583		Lower −0.263 Upper 0.0923	0.918	0.346		Lower −0.147 Upper 0.2756	1.066	0.552		Lower −0.410 Upper 0.12094	0.865	0.286		Lower −0.419 Upper 0.1907	0.892	0.463	
Intercept	Lower 2.9164 Upper 2.965	18.93	<0.001		Lower 2.5375 Upper 2.596	13.02	<0.001		Lower 2.05433 Upper 2.128	8.09	<0.001		Lower 1.670 Upper 1.759	5.554	<0.001		Lower 1.2720 Upper 1.380	3.77	<0.001	
**Religion**																				
Non-muslim–Muslim	Lower 0.0914 Upper 0.219	1.17	<0.001	0.00967	Lower −0.0616 Upper 0.101	1.02	0.634	4.21e-4	Lower −0.00271 Upper 0.197	1.10	0.057	0.00668	Lower −0.132 Upper 0.120	0.994	0.923	1.13e-5	Lower 0.0873 Upper 0.364	1.25	0.001	0.0110
Intercept	Lower 3.032 Upper 3.108	21.541	<0.001		Lower 2.4695 Upper 2.570	12.43	<0.001		Lower 2.0418 Upper 2.1657	8.20	<0.001		Lower 1.719 Upper 1.8642	6.000	<0.001		Lower 1.463 Upper 1.627	4.689	<0.001	
**Marital status**																				
Single–married	Lower −0.207 Upper −0.113	0.852	<0.001	0.0193	Lower 0.0103 Upper 0.130	1.07	0.022	0.00993	Lower −0.0742 Upper 0.0745	1.00	0.997	2.44e-8	Lower −0.203 Upper −0.0257	0.892	0.011	0.00766	Lower −0.386 Upper −0.180	0.753	<0.001	0.0320
Intercept	Lower 2.757 Upper 2.829	16.33	<0.001		Lower 2.5161 Upper 2.5975	12.89	<0.001		Lower 2.067 Upper 2.1683	8.311	<0.001		Lower 1.5475 Upper 1.678	5.02	<0.001		Lower 1.039 Upper 1.206	3.07	<0.001	
**Qualification**																				
Masters–bachelors	Lower 0.159 Upper 0.267	1.24	<0.001	0.0816	Lower −0.0639 Upper 0.0657	1.00	0.978	0.00406	Lower−0.114 Upper 0.0487	0.968	0.430	0.00126	Lower 0.0522 Upper 0.251	1.16	0.003	0.0216	Lower 0.136 Upper 0.383	1.30	<0.001	0.0861
Ph.D–bachelors	Lower 0.274 Upper 0.398	1.40	<0.001		Lower −0.0247 Upper 0.1286	1.05	0.184		Lower −0.120 Upper 0.0766	0.979	0.667		Lower 0.0870 Upper 0.320	1.23	<0.001		Lower 0.348 Upper 0.620	1.62	<0.001	
Post doc–bachelors	Lower 0.344 Upper 0.494	1.52	<0.001		Lower −0.0648 Upper 0.1317	1.03	0.504		Lower −0.132 Upper 0.1171	0.993	0.908		Lower 0.0596 Upper 0.353	1.23	0.006		Lower 0.436 Upper 0.759	1.82	<0.001	

## Discussion

Toxoplasmosis is a major global zoonotic disease that has a deleterious effect on human health, with severe consequences in immunocompromised, pregnant women (10). Consumption of contaminated raw meat, water, fruits, and vegetables; contact with cats; and exposure to soil contaminated with cat feces are the main transmission routes (11). Out of 400 participants, 240 (60%) were aware of toxoplasmosis. Similar findings have been reported in Northeast Ethiopia (1).

Our study findings revealed that 87.3 and 85.5% of participants washed their hands after gardening and changing the cat litter, respectively. Additionally, 89% of participants thoroughly cooked meat prior to consumption, and 86.3% avoided drinking raw milk. A study from Ethiopia reported that among pregnant women, 77.6% washed their hands after gardening, 64.7% washed their hands after changing the cat litter, and 62.2% washed their hands after handling raw meat. Furthermore, 85.9% of the pregnant women reported that they did not avoid drinking untreated water (1). In our study, 80% of participants considered toxoplasmosis to be a dangerous disease, and 33.5% reported that they had not consumed undercooked meat. In contrast, a study reported that 51.4% of participants did not consider toxoplasmosis to be a severe disease. Additionally, 48% individuals were unsure whether toxoplasmosis was spread via consumption of inadequately washed vegetables (30). Our study showed that 81.8% of participants washed their kitchen utensils after contact with raw meat or unwashed fruits and vegetables. Similar findings were obtained in Brazil, where 24.7% of pregnant women reported washing kitchen utensils (31). Approximately 30% of the participants did not allow anyone else to change the cat litter box. Similar findings have been reported in a study conducted in Northeast Ethiopia where 51.3% women responded that they did not allow someone else to change the cat litter box (1).

Most of the participants (76.8%) reported that toxoplasmosis was acquired by consuming raw/undercooked meat. These findings were consistent with those of a study carried out in Mexico, where more than half of the respondents correctly defined the routes of transmission: (1) consumption of raw or undercooked foods, unwashed fruits and vegetables and (2) direct contact with cats (32). In our study, 69.5% of participants considered blood transfusion to be a cause of toxoplasmosis. In one of the surveys, 27.7% of the participants did not assume that blood transfusion could spread toxoplasmosis, and 38.5% believed that it could be transmitted from the mother to her fetus (33). Approximately 68.3% of participants responded that gardening without gloves could be a transmission source of toxoplasmosis. In a study conducted in the US, 29% of the participants thought that toxoplasmosis could be transmitted by gardening without gloves (34). Our study findings showed that immunocompromised pregnant women had a high risk of toxoplasmosis similar to the findings of Desta who reported there is a high risk of toxoplasmosis in immunocompromised, pregnant women (77.9%) (1). The majority (58%) of participants reported that toxoplasmosis is a zoonotic infection. A previous study reported that 33.82% of participants were aware that toxoplasmosis is a zoonotic disease (1).

### Strength, Limitations, and Future Recommendations

The limited amount of knowledge about toxoplasmosis emphasized to provide and promote health education regarding toxoplasmosis especially awareness regarding transmission of disease in the pregnant women. It is important to improve primary health care system of the country for the better control, management, and prevention of the disease. Moreover, it is stressed that in the study population to commence health education and awareness campaigns for the community and to design relevant policies for the guidance of the government and stakeholders to reduce the risk of disease. In the study design, the use of close questionnaire is one of the limitation, where free form response was not allowed. In our study included the participants from university which is not representative of the situation of whole country. The strength of the study is maximum number of female participants and preliminary study on the knowledge about toxoplasmosis among university students in Pakistan.

## Materials and Methods

### Study Area

We conducted a cross-sectional analysis in Islamabad and Rawalpindi district of Punjab, Pakistan, also known as twin cities. The terrain consists of plains and mountains in the metropolitan area of Islamabad and Rawalpindi. In the mountainous terrain of Margala hills is the northern part of the metropolitan area, while Rawalpindi is situated on the Pothohar plateau (35).

### Participants

The study participants included students from universities of the twin cities that were enrolled in different degree programs (Bachelors, Masters, Ph.D., and Post doc). The sample size was calculated using Raosoft software (http://www.raosoft.com/samplesize.html; 5% margin of error, 95% confidence level, and 50% response distribution). Four hundred questionnaires were randomly distributed and filled by the participants. We collected data from July through December 2020.

### Sample Size

A questionnaire was designed to access the knowledge, attitude, practices, risk factor and one health regarding toxoplasmosis. A total of 400 questionnaires were administrated. The questionnaire was categories into the following sections as demography (*n* = 17), knowledge (*n* = 34), attitude (*n* = 19), practices (*n* = 11), risk factors (*n* = 8), and one health (*n* = 7).

### Data Collection

We developed a structured questionnaire to collect the data. After obtaining verbal informed consent from the participants, we conducted interviews. A team was trained for interviews, data collection, and record keeping. A supervisor routinely coordinated the interview process to ensure adequate data collection and record maintenance. The purpose of study was explained to the participants. The questionnaire consisted of six sections. The first section was on the socio-demographics of the participants. The second section was on the knowledge on toxoplasmosis, including common signs, symptoms, and diagnostic tests used for toxoplasmosis. The third section was on the attitudes and perceptions toward toxoplasmosis. The fourth section was on practices performed when toxoplasmosis was either suspected or diagnosed. The fifth section was on major risk factors of the disease, and the sixth section was on One Health questions regarding toxoplasmosis.

### Statistical Analysis

We generated a database using Excel (Microsoft, Redmond, WA, USA) and calculated basic frequencies. We used descriptive statistics to initially analyze the data and classified the variables into independent and dependent variables. We performed statistical analysis using Jamovi software (version 1.6.7; https://www.jamovi.org) to observe the factors involved in the occurrence of toxoplasmosis. The relationship between various factors influencing knowledge, attitudes, and practices was analyzed. For data analysis, we used Chi square test, one-way analysis of variance (ANOVA), and log-linear regression.

## Conclusions

There is a low level of knowledge and awareness regarding toxoplasmosis among males. Therefore, there should be awareness programs to educate individuals about the risks of this deadly disease and to provide information on the major routes of transmission. Our study highlights the need of toxoplasmosis awareness to reduce the burden and economic impact of the disease.

## Data Availability Statement

The original contributions presented in the study are included in the article/supplementary material, further inquiries can be directed to the corresponding author/s.

## Ethics Statement

The studies involving human participants were reviewed and approved by the Ethics Committee of COMSATS University. The patients/participants provided their written informed consent to participate in this study.

## Author Contributions

HA and KS designed and supervised the study. TM and KS performed the data collection. KS, SS, SA, MA, HA, and JC conducted statistical and data analysis. SN drafted the manuscript. SS and JC performed critical revisions. All authors read and approved the final manuscript.

## Funding

This study was supported by the Fifth Round of Three-Year Public Health Action Plan of Shanghai (Grant No. GWV-10.1-XK13 to JC), the National Natural Science Foundation of China (Grant Nos. 81772225 and 81971969 to JC), and the Key Laboratory of Parasite and Vector Biology, National Health Commission of People's Republic of China (Grant No. WSBKFKT2017-01). The funders had no role in the study design, the data collection and analysis, the decision to publish, or the preparation of the manuscript.

## Conflict of Interest

The authors declare that the research was conducted in the absence of any commercial or financial relationships that could be construed as a potential conflict of interest.

## Publisher's Note

All claims expressed in this article are solely those of the authors and do not necessarily represent those of their affiliated organizations, or those of the publisher, the editors and the reviewers. Any product that may be evaluated in this article, or claim that may be made by its manufacturer, is not guaranteed or endorsed by the publisher.

## Abbreviations

WHO: World Health Organization
NZDs: Neglected Zoonotic Diseases
*T. gondii*: *Toxolasma gondii*
DALYs: Disability Adjusted Life Years.
